# Patient Activation, Social Support, Physician Trust, and Shared Dialysis Decision-Making: A Cross-Sectional Investigation

**DOI:** 10.1016/j.xkme.2025.101014

**Published:** 2025-04-19

**Authors:** Fahad Saeed, Basil S. Kazi, Musaib Syed, Kevin A. Fiscella, Paul R. Duberstein

**Affiliations:** 1 Department of Medicine, Division of Nephrology, Division of Palliative Care, University of Rochester School of Medicine and Dentistry, Rochester, NY; 2 Department of Internal Medicine, University of Illinois at Chicago, Chicago, Illinois; 3 Department of Family Medicine and Center for Center for Communication and Disparities Research, University of Rochester School of Medicine and Dentistry, Rochester, NY; 4 Department of Public Health, University of Rochester School of Medicine and Dentistry, Rochester, NY; 5 Department of Health Behavior, Society and Policy, Rutgers School of Public Health, Piscataway, NJ; 6 Department of Biomedical Sciences, Rutgers School of Graduate Studies, Newark, NJ

**Keywords:** Dialysis decision-making, patient activation, shared decision-making, social support, physician trust

## Abstract

**Rationale & Objective:**

People undergoing maintenance dialysis often lack essential information about kidney therapy options. Therefore, nephrologists must involve patients and families in shared decision-making (SDM). In this investigation, we hypothesized that patient activation, social support, and physician trust would be associated with patient-reported SDM.

**Study Design:**

A cross-sectional survey.

**Methods:**

We surveyed hospitalized individuals receiving maintenance dialysis. Participants completed the 9-item SDM questionnaire (SDM-Q-9) and the Patient Activation Measure-13, the Multidimensional scale of perceived social support, and the Primary Care Assessment Survey Trust Subscale.

**Analytic Approach:**

We used descriptive statistics to present demographics. We included patient demographics in multivariable linear regression models predicting SDM scores.

**Results:**

Of the 223 respondents, 222 patients completed SDM-Q-9, 54% were ≥65 years old, with 47% being woman. In addition, 37% self-identified as African American, 48% had an education level at ≤high school, and 54% reported an annual household income of ≤$20,000. The SDM-Q-9 scores (n = 222) had a mean of 57.49 (SD = 27.07), median 58 (IQR = 38-82), and ranged from 0 to 100. The mean patient activation was 53.5 ± 16.5 (min 24, max 100), social support 61.2 ± 24 (min 0, max 100), and physician trust 59.29 ± 21.6 (min 0, max 100). In separate multivariable linear regression models, higher patient activation (1-point patient activation measure increase per 0.48-unit SDM increase; 95% CI, 0.24-0.73), higher social support (1-point increase per 0.28 SDM increase; 95% CI, 0.11-0.45), and greater physician trust (0.31-point increase per 1-point SDM increase; 95% CI, 0.12-0.49) were significantly associated with greater SDM.

**Limitations:**

The study was a cross-sectional investigation.

**Conclusions:**

Our findings indicate a significant association between SDM and patient activation, social support, and physician trust. Future research with individuals actively considering kidney therapy options should prospectively explore the relationship between patient activation, social support, physician trust and SDM.

## Plain Language Summary

There is limited literature on the association between patient activation, social support, and physician trust with patient-reported shared dialysis decision-making. Therefore, we examined this association in a convenience sample of 222 people receiving maintenance dialysis. We found that patient activation, social support, and physician trust were independently associated with shared dialysis decision-making. Future prospective studies are needed to explore the relationship between patient activation, social support, physician trust, and shared dialysis decision-making among individuals actively considering kidney therapy options.

The American Society of Nephrology and the Renal Physicians Association have endorsed nephrologists involving patients and families or other caregivers in shared decision-making (SDM) regarding kidney therapy (KT) choices.^1^^,^^2^ The SDM represents a collaborative approach wherein both patients and clinicians jointly reach treatment decisions.^3^ Ideally, the process of shared KT decision-making should encompass a comprehensive exploration of all available KT options, such as kidney transplantation, peritoneal dialysis, hemodialysis, conservative kidney management, time-limited dialysis trials, palliative dialysis, and even the possibility of deciding not to decide.^4^^,^^5^ The clinician’s role also extends to presenting the merits and drawbacks of each option, understanding patient preferences, and aiding the patient in selecting an option that aligns with their preferences. Consequently, the integration of SDM into the decision-making process holds pivotal significance, being associated with high satisfaction with the decision-making process,^6^ improved disease-specific health-related quality of life,^7^ and reduced health care expenditures.^8^ Remarkably, the absence of SDM is linked to elevated odds of both poor physical and mental health and increased emergency department utilization.^9^

Nonetheless, the practice of SDM in individuals with advanced kidney disease facing dialysis decisions remains relatively infrequent.^10^ In a study involving 350 individuals with kidney disease, only 41% perceived their treatment choice as a mutual decision with their clinician.^11^ Expectedly, the majority grapple with substantial decisional conflicts concerning the choices at hand and the potential ramifications on their daily lives.^12^ Many do not recall being presented with alternatives to dialysis, such as conservative kidney management, and some even describe undue influences from clinicians and family members, nudging them toward selecting dialysis.^13^^,^^14^ Consequently, nearly one-fifth of patients later regret their decision to initiate dialysis.^14^ Intriguingly, patients are more prone to regret their dialysis decision if influenced by physicians and family members, whereas those engaging in prognostic discussions with their physician are less likely to experience regret.^14^ Simply put, SDM remains largely absent from KT decisions.

Shared decision-making is, undeniably, a shared, although not equal, responsibility involving both clinicians and patients. Clinician-related factors, such as previous training in communication skills and SDM,^15^ preference for medical paternalism,^7^ and the absence of financial incentives for SDM or CKM^16^ may collectively discourage clinicians from engaging patients in SDM. Similarly, patient-related factors may also diminish patient participation in SDM. A study involving 63,931 participants found that certain patients were less likely to experience SDM: non-White race, lower educational attainment, low income, unmarried status, and lack of insurance coverage.^9^ Similarly, a review of multiple patient surveys identified older age, lower educational attainment, and man gender as predictors of a lesser inclination toward SDM and a higher preference for assuming a passive role in the clinician-patient relationship.^17^ Notably, the extent to which patients engage in the decision-making process can also influence physicians’ decision-making style, potentially leading them to adopt a more paternalistic approach.^18^

Although previous studies have examined demographic variables associated with SDM, this study examines psychosocial variables. Drawing on previous research documenting the salutary effects of patient activation,^19^ social support,^20^^,^^21^ and physician trust,^22^ we hypothesized that each of these variables would be associated with SDM. Indeed, a previous investigation involving 1,222 patients with diabetes or cardiovascular disease revealed a noteworthy positive association between patient activation and increased engagement in SDM.^19^ Moreover, this heightened engagement in SDM was reciprocally linked to elevated patient activation levels 1 year later.^19^ Furthermore, the presence of robust support from a caring family member can significantly enhance a patient’s self-assuredness in navigating intricate decision-making processes and activate them to participate more actively in the overall decision-making experience.^20^^,^^21^ In a study encompassing individuals experiencing chronic pain, a positive correlation was established between the availability of social support and patient activation.^23^ In addition, an investigation involving 509 patients with rheumatoid arthritis shed light on the critical role of physician trust in the context of SDM: low levels of physician trust were associated with suboptimal SDM, underscoring the pivotal importance of the physician-patient relationship as a predictor of effective SDM.^22^

In this current cross-sectional investigation, we aimed to examine the association of patient activation, social support, and physician trust with SDM. Drawing from previous literature showing a positive association between patient activation^19^ and social support with SDM,^20^^,^^21^ and a negative association between low physician trust and SDM in non-renal populations,^22^ we hypothesized that higher scores on the Patient Activation Measure-13 (PAM-13), Multidimensional Scale of Perceived Social Support (MSPSS), and Primary Care Assessment Survey (PCAS) trust subscale will be associated with greater scores on the 9-item SDM questionnaire (SDM-Q-9).

## Methods

Deploying convenience sampling methodology, we surveyed hospitalized patients at a single academic medical center in Rochester, New York from June 2019, to March 2020. Adult hospitalized patients receiving maintenance dialysis who were ≥18 years of age and spoke English were eligible to participate. Although we enrolled hospitalized patients from a single, 14 bed, in-patient dialysis unit, these patients were receiving outpatient dialysis at clinics located throughout the Greater Rochester area. We decided to study only hospitalized individuals because of sampling convenience and difficulty approaching patients being dialyzed at industry-owned dialysis units. To screen for eligibility, a member of the research team first approached the dialysis team caring for these patients and asked if the patient would be physically and cognitively able to participate in the study. Notably, the study team did not approach patients who were too sick to participate as reported by the clinician caring for the patient. A member of the study team explained the purpose of the study to eligible patients. Those who were able to explain back the purpose of the study and sign the written consent form were enrolled in the study. Patients had the option of either answering the questions themselves or have a study team member orally administer the survey. Patients were offered a chance to enter a raffle to win a $50 gift card. A total of 4 gift cards were available for the raffle. The institutional review board at the University of Rochester Medical Center approved this study.

### Outcome Variable

The primary outcome variable (used as a continuous variable) is the SDM-Q-9 scale to ask about dialysis decision-making, a 9-item scale (Cronbach’s α  =  0.94).^24^ The SDM-Q-9 captures information about different steps of decision-making, such as the clinician talking about the need for decision-making, presenting different treatment choices, explaining the pros and cons of each choice, incorporating patient preference into the final decision and providing anticipatory guidance about the next steps. Response options to 9 questions range from completely disagree, strongly disagree, somewhat disagree, somewhat agree, strongly agree, or completely agree.^24^ Higher scores on the SDM scale reflect higher patient engagement in the SDM process. This scale has previously been used to assess shared dialysis decision-making.^25^

### Independent Variables

Patient Activation Measure-13 (used as continuous variable) is a 13-item scale (Cronbach’s α  =  0.86) to measure patient activation. It captures information about the degree of patient’s confidence, skills, and disease knowledge to self-manage their condition.^26^ Response options include completely disagree, strongly disagree, agree, strongly agree, strongly agree, and not applicable.^26^ This scale has previously been used in the dialysis population.^27^ The PAM-13 scale can be categorized into 4 levels: level 1 (score 0-47), level 2 (score 47.1-55.1), level 3 (score 55.2-72.4), and level 4 (score 72.5-100).^26^ Higher scores on the PAM-13 scale mean higher patient activation.

Multidimensional Scale of Perceived Social Support (used as continuous variable) is a scale (Cronbach’s α =  0.88) to assess social support.^28^ This scale has an11-item subscale to assess support from significant other, family, and friends. Response options on a 7-point Likert scale range from very strongly disagree to very strongly agree. Higher scores mean greater social support.

Primary Care Assessment Survey trust subscale (used as continuous variable) is an 8-item subscale to measure various aspects of patient trust in their doctor, such as patient’s sense of psychological safety, physician’s integrity, and professional competence.^29^ Response options range from strongly disagree, disagree, slightly disagree, slightly agree, agree to strongly agree. A higher score on the scale means more trust in the physician.

Covariates included patient demographics, age (≥65 vs <65 to categorize older and younger patients), self-identified gender (women vs men vs transgender vs other), self-identified race (White vs Black vs others, such Asian, Native Hawaiian or other Pacific Islander, American Indian or Alaska Native, or missing), education (above high school vs high school or below), annual household income (≤$20,000 vs >$20,000 to categorize poverty),^30^ and time on dialysis. These patient level variables were selected because of the potential relationship of man gender, Black race, low education, and low income with the degree of engagement in SDM.^9^^,^^17^

### Analyses

Scores for the SDM-Q-9 scale^24^ and 3 key independent variables, that is, PAM-13,^26^^,^^27^ MSPSS,^28^ and PCAS trust subscale^29^ were calculated. We further transformed the scores for SDM-Q-9 scale,^24^ PAM-13,^26^^,^^27^ MSPSS,^28^ and PCAS trust subscale^29^ on a theroretical range of 0-100. Missing responses (n = 1) for SDM-Q-9 were completely excluded from analyses.

We checked the linear regression assumptions, including checking for linearity in the relationship between the dependent and independent variables, assessing multicollinearity, examining the normality of the collected data, and ensuring homoscedasticity by checking for homogeneously distributed variance in the data. Correlations between the SDM and the 3 key independent variables, using Pearson Correlations were also examined. Because of the presence of collinearity between all these variables, we built 3 separate linear regression models to establish the association of 3 independent variables and 6 covariates with the dependent variable of SDM-Q-9. A complete case analyses approach was employed and missing variables were excluded from our analyses. The final regression models had 175 observations. Three additional multivariable linear regression models were built using similar methods but using a different cut-off for education, that is, more than or equal to college level education versus less than college level education. We also built a fourth multivariable regression model after categorizing PAM into levels 1-4. We then further developed 3 additional regression models after imputing missing values: the mode for categorical variables and the mean for continuous variables.

## Results

Of the 380 patients approached, 223 (59% response rate) agreed to participate and 222 provided data on SDM-Q-9 scale. Table 1 shows the demographics of the respondents. Of these, 54% (n = 120) were 65 years or older, with 47% (n = 104) being woman. In addition, 37% self-identified as African American (n = 82), 48% (n = 105) had an educational attainment level at or below high school, and 54% (n = 119) reported an annual household income of $20,000 or less. The SDM-Q-9 scores (n = 222) had a mean of 57.49 (SD = 27.07), a median of 58 (IQR = 38-82), and ranged from 0 to 100. We did not intentionally exclude individuals receiving peritoneal dialysis. However, our sample only included individuals undergoing hemodialysis, likely due to the smaller population of patients receiving peritoneal dialysis in the Greater Rochester area. Table S1 presents the demographics of participants, using the SDM cut-off at the median value.

**Table 1:** Baseline Characteristics

Parameter Statistic	Total (N = 223)
Age category, n (%)	
˂65	92 (41.3%)
≥65	120 (53.8%)
Missing	11 (4.9%)
Gender, n (%)	
Woman	104 (46.6%)
Man	105 (47.1%)
Transgender	3 (1.3%)
Missing	11 (4.9%)
Race, n (%)	
White	91 (40.8%)
Black/African American	82 (36.8%)
Other	40 (17.9%)
Missing	10 (4.5%)
Education, n (%)	
Greater than high school	105 (47.1%)
High school or Less	106 (47.5%)
Missing	12 (5.4%)
Annual household income, n (%)	
≤$20,000	119 (53.4%)
>$20,000	75 (33.6%)
Missing	29 (13.0%)
Time on dialysis (y)	
n	205
Mean ± SD	3.16 ± 2.37
Median (Q1-Q3)	3 (1-4)
Min; Max	0; 13
SDM-Q-9	
n	222
Mean ± SD	57.49 ± 27.07
Median (Q1-Q3)	58 (38-82)
Min; max	0; 100
Patient activation measure levels	
Level 1	104 (46.6%)
Level 2	28 (12.6%)
Level 3	50 (22.4%)
Level 4	33 (14.8%)
Missing	8 (3.6%)
Patient activation measure	
n	215
Mean ± SD	53.50 ± 16.46
Median (Q1-Q3)	49 (41-66)
Min; max	24; 100
Multidimensional scale of perceived social support	
n	220
Mean ± SD	61.2 (24.32)
Median (Q1-Q3)	56 (45-86)
Min; max	0; 100
Primary care assessment survey (PCAS)	
n	215
Mean ± SD	59.29 ± 21.6
Median (Q1-Q3)	58 (45-78)
Min; max	0; 100

Because of issues of collinearity between PAM-13, PCAS trust subscale, and social support scale, 3 separate linear regression models were built. The first model (Table 2) examined the association of PAM-13 with SDM-Q-9 while accounting for age, gender, race, education, income, and dialysis duration. A positive correlation of higher PAM-13 (0.48 [95% CI, 0.24-0.73]; *P* ≤ 0.001) scores with greater odds of high SDM-Q-9 scores was observed.

**Table 2:** Multivariable Linear Regression Models Examining the Association of Predictors With SDM

Variable	Model 1. Patient Activation	Model 2. Social Support	Model 3. Physician Trust
	Estimate (95% CI)	Estimate (95% CI)	Estimate (95% CI)
Age category (y)			
<65	Reference		
≥65	9.25 (1.27-17.22)	9.05 (1.03-17.07)	8.97 (0.95-17.00)
Sex			
Woman	Reference		
Man	2.04 (−5.85 to 9.94)	2.58 (−5.35 to 10.50)	3.20 (−4.70-11.09)
Race			
White	Reference		
Black/African American	−5.33 (−14.09 to 3.43)	−6.00 (−14.80 to 2.81)	−5.24 (−14.10 to 3.61)
Other	3. 02 (−7.94 to 13.97)	1.33 (−9.7 to 12.38)	3.40 (−7.67 to 14.46)
Education			
≥ High school	Reference		
< High school	0.72 (−7.29 to 8.73)	1.21 (−6.88 to 9.29)	−0.34 (−8.40 to 7.73)
Income			
>$20,000	Reference		
≤$20,000	−1.82 (−9.90 to 6.26)	−2.12 (−10.26 to 6.03)	−2.22 (−10.38 to 5.93)
Time on dialysis (y)	0.96 (−0.84 to 2.76)	0.77 (−1.05 to 2.59)	1.27 (−0.55 to 3.09)
Patient activation	0.48 (0.24-0.73)^a^	–	–
Social support	–	0.28 (0.11-0.45)^a^	–
Physician trust	–	–	0.31 (0.12-0.49)^a^

a Denotes *P* ≤ 0.001.

The second linear regression model (Table 2) examined the association of MSPSS with SDM-Q-9 while accounting for other covariates. A positive correlation of higher MSPSS scores (0.28 [95% CI, 0.11-0.45]; *P* ≤ 0.001) with greater odds of having higher scores on SDM-Q-9 was observed.

The third linear regression model (Table 2) examined the association of PCAS trust subscale with SDM-Q-9 while accounting for age, gender, race, education, and income. In this model, the association of PCAS trust subscale with SDM-Q-9 scores was significant (0.31 [95% CI, 0.12-0.49]; *P* ≤ 0.001).

We conducted 3 additional sensitivity analyses. First, we used a different cut-off for educational attainment equal to or above college level education vs below, and findings are broadly consistent with those in Table 2 (data not shown). Second, we categorized PAM into 4 levels.^26^ The association of PAM with SDM remained significant (Table S2). Third, we imputed missing values. The findings were broadly consistent with those reported in Table 2.

## Discussion

In this investigation of hospitalized people receiving maintenance dialysis, higher patient activation, social support, and physician trust were associated with greater shared KT decision-making.

Three main findings merit discussion. First, patients with higher scores on the PAM were more likely to report engagement in the SDM process. This finding is consistent with previous literature in a non-dialysis population showing an association of patient activation with SDM.^19^ A relevant study from the dialysis literature showed an association of decision self-efficacy, an aspect of patient activation, with reduced decisional conflict.^31^ Strikingly, consistent with the previous literature in a non-dialysis population, although the decision to initiate dialysis in this cohort of acutely ill hospitalized patients was made in the past, the association of patient activation with SDM still remained significant.^32^ Others have shown the role of patient activation in promoting desirable health behaviors, such as routine diabetic foot check, regular exercise, blood pressure control and cholesterol levels,^33^, ^34^, ^35^ cancer care for minority populations,^36^^,^^37^ post stroke care,^38^ and cardiovascular risk reduction.^39^ Our study adds to the literature by showing a positive association of patient activation with shared KT decision-making.

Second, patients with higher social support scores were more likely to report being engaged in the SDM process. This finding is also consistent with previous literature showing association of social support with patient’s active role in medical decision-making.^40^ It is possible that people with higher social support are better able to cope with medical decisions and navigate through complex decision-making processes. For example, one study showed that in patients with advanced kidney disease facing dialysis decisions, higher social support was positively correlated with higher sense of hope and reduced decisional conflict.^20^ It is also plausible that with better social support, patients are able to reflect, weigh the pros and cons, and feel validated in their decisions, a concept termed as shared mind by Epstein et al.^21^ However, undue influences from family members have also been shown to potentially undermine a patient’s sense of agency and promote dialysis regret.^14^ Future studies investigating the influence of social network characteristics on dialysis decision-making are needed.

Third, perceived trust in the physician was significantly associated with greater reported SDM. This finding is consistent with previous literature showing associations between physician trust and SDM.^23^^,^^28^ This finding needs to interpreted with caution because we asked patients about their trust of physicians in general, not the physician who helped them make dialysis decision or the physician who was caring for them in the hospital or their primary care doctor.

This study has several strengths and limitations. To the best of our knowledge, the association of SDM with patient activation, physician trust, and social support has not been previously reported in a sample of hospitalized persons receiving dialysis. However, this study is limited by its cross-sectional design, with study sample recruited from a single academic center affecting its generalizability. We did not maintain records of patient’s outpatient dialysis unit and patients who underwent oral survey administration versus those who independently completed the surveys, and those deemed ineligible to participate due to their health status by their clinician or individuals who could not complete the informed consent process. Further, study patients were hospitalized and ill, which may have influenced their affective state and responses to questions about recall of dialysis decision-making process or sense of activation or physician trust. Moreover, cross-sectional studies that use only one method for assessing constructs of interest—in our case, self-report—are subject to the common method bias.^41^ Observed estimates are thus partially attributable to shared method variance, potentially leading to spuriously inflated estimates. Finally, we were unable to account for clinician communication and practice style in our model and asked patients about their shared dialysis decision-making process in general, not with their nephrologist or primary care clinician.

This study has several research and clinical implications. The study findings call for further research efforts to examine prospectively the role of patient activation, social support, and physician trust in dialysis decision-making and, ultimately, randomized trials that examine the effect of interventions to increase patient activation on SDM around ESKD treatment options. Importantly, such research efforts will need to be clinically complemented by clinician training in communication and SDM skills^42^ and family members’ engagement in the KT decision-making process. Currently, many family members of patients with kidney disease are not invited by health care teams to participate in these important decisions.^43^ Not surprisingly, many family members have poor understanding of the patient’s prognosis and few understand the disease trajectory.^44^ Future studies to examine physician trust and activation of family members of people facing dialysis decisions and its association with improving the KT decision-making process may also be helpful.

In summary, we found that patient activation, physician trust, and social support were positively associated with SDM. Our findings call for future research efforts to study patient activation and its putative causal role in SDM process for people with chronic kidney disease.
