# Supplementary material for: Patient Activation, Social Support, Physician Trust, and Shared Dialysis Decision-Making: A Cross-Sectional Investigation
Source: Kidney Med. 2025 Apr 19;7(6):101014. doi: 10.1016/j.xkme.2025.101014 (PMC12152607; doi:10.1016/j.xkme.2025.101014)
Supplement: Supplementary File (PDF) — Tables S1, S2. [file mmc1.pdf]

**Table S1. Baseline Characteristics of the participants with SDM cut-off at the Median Value**			
**Parameter Statistic**	**SDM Score ≤58 (N=114)**	**SDM Score >58 (N=108)**	**Total (N=222)**
**Age Category, n (%)**			
< 65	50 (43.9%)	42 (38.9%)	92 (41.4%)
≥ 65	56 (49.1%)	64 (59.3%)	120 (54.1%)
Missing	8 (7.0%)	2 (1.9%)	10 (4.5%)
**Gender, n (%)**			
Female	58 (50.9%)	46 (42.6%)	104 (46.8%)
Male	48 (42.1%)	57 (52.8%)	105 (47.3%)
Transgender	2 (1.8%)	1 (0.9%)	3 (1.4%)
Missing	6 (5.3%)	4 (3.7%)	10 (4.5%)
**Race, n (%)**			
White	45 (39.5%)	46 (42.6%)	91 (41.0%)
Black/African American	45 (39.5%)	37 (34.3%)	82 (36.9%)
Other	18 (15.8%)	22 (20.4%)	40 (18.0%)
Missing	6 (5.3%)	3 (2.8%)	9 (4.1%)
**Education, n (%)**			
Greater than high school	48 (42.1%)	57 (52.8%)	105 (47.3%)
Highschool or less	58 (50.9%)	47 (43.5%)	105 (47.3%)
Missing	8 (7.0%)	4 (3.7%)	12 (5.4%)
**Income, n (%)**			
≤ $20,000	58 (50.9%)	61 (56.5%)	119 (53.6%)
≥$20,001	40 (35.1%)	34 (31.5%)	74 (33.3%)
Missing	16 (14.0%)	13 (12.0%)	29 (13.1%)
**Patient Activation Measure Levels**			
Level 1	69 (60.5%)	35 (32.4%)	104 (46.8%)
Level 2	11 (9.6%)	17 (15.7%)	28 (12.6%)
Level 3	21 (18.4%)	29 (26.9%)	50 (22.5%)
Level 4	8 (7.0%)	25 (23.1%)	33 (14.9%)
Missing	5 (4.4%)	2 (1.9%)	7 (3.2%)
**Time on Dialysis (Years)**			
n	103	102	205
Mean (SD)	3.07 (2.04)	3.24 (2.67)	3.16 (2.37)
Median	3	3	3
Q1; Q3	2; 4	1; 4	1; 4
Min; Max	0; 12	0; 13	0; 13

**Primary Care Assessment Survey (PCAS)**			
n	108	107	215
Mean (SD)	29.47 (7.77)	33.98 (8.93)	31.72 (8.65)
Median	29	33	31
Q1; Q3	25; 33	28; 42	26; 39
Min; Max	8; 48	15; 48	8; 48
**Trust Transformed Score**			
n	108	107	215
Mean (SD)	53.68 (19.42)	64.95 (22.32)	59.29 (21.62)
Median	53	63	58
Q1; Q3	43; 63	50; 85	45; 78
Min; Max	0; 100	18; 100	0; 100
**Multidimensional Scale of Perceived Social Support**			
n	112	108	220
Mean (SD)	54.07 (21.81)	68.63 (24.65)	61.22 (24.32)
Median	52	67	56
Q1; Q3	42; 66	48; 92	45; 86
Min; Max	0; 100	0; 100	0; 100

Table S2. Multivariate Linear Regression model examining the association of four levels PAM with SDM

Parameter Statistic	Estimate	95% Confidence Interval	P-value
**Age Category**			
<65	Reference		
≥65	9.22	1.19, 17.25	0.025
**Gender**			
Female	Reference		
Male	2.22	-5.65, 10.10	0.578
**Race**			
White	Reference		
Black/African American	-5.02	-13.79, 3.74	0.260
Other	3.54	-7.47, 14.55	0.527
**Education**			
≥ College	Reference		
≤ Highschool	0.46	-7.54, 8.47	0.909
**Income**			
>$20,000	Reference		
≤$20,000	-1.10	-9.27, 7.06	0.790
**Time on Dialysis (Years)**	0.84	-0.96, 2.65	0.358
**Patient Activation Measure Level**	Reference		
Level 1	10.42	-2.26, 23.09	0.107
Level 2	12.98	3.08, 22.88	0.011
Level 3	22.45	11.05, 33.85	<0.001
Level 4			

1) Dependent variable SDM score.
2) Independent variables are Pam Levels, Dialysis Years, Age Category, Gender, Race, Education, Income
